# Effects of Tocilizumab Therapy on Circulating B Cells and T Helper Cells in Patients With Neuromyelitis Optica Spectrum Disorder

**DOI:** 10.3389/fimmu.2021.703931

**Published:** 2021-07-29

**Authors:** Ye Liu, Huiming Zhang, Tian-Xiang Zhang, Meng Yuan, Chen Du, Pei Zeng, Zhenning Huang, Dongmei Jia, Guili Yang, Fu-Dong Shi, Chao Zhang

**Affiliations:** ^1^Department of Neurology, Tianjin Medical University General Hospital, Tianjin Neurological Institute, Key Laboratory of Post-Trauma Neuro-Repair and Regeneration in Central Nervous System, Ministry of Education, Tianjin Key Laboratory of Injuries, Variations and Regeneration of Nervous System, Tianjin, China; ^2^Department of Neurology, The Third People’s Hospital of Datong, Datong, China; ^3^Jing-Jin Center for Neuroinflammation, China National Clinical Research Center for Neurological Diseases, Beijing Key Laboratory of Translational Medicine for Cerebrovascular Disease, Beijing Tiantan Hospital, Capital Medical University, Beijing, China

**Keywords:** NMOSD, tocilizumab, B cells, T helper cells, PD-1, PD-L

## Abstract

Tocilizumab, a humanized anti-IL-6 receptor monoclonal antibody, showed its therapeutic efficacy on neuromyelitis optica spectrum disorder (NMOSD). To assess the immunological effects of this drug on B cells, follicular T helper (Tfh) cells, and peripheral T helper (Tph) cells in patients with NMOSD, peripheral B cell and Tfh cell phenotypes were evaluated in 26 patients with NMOSD before and after tocilizumab treatment by nine-color flow cytometry, as well as the expression of costimulatory and co-inhibitory molecules on B cells. Results showed that the frequency of CD27^+^IgD^−^ switched memory B cells, CD27^-^IgD^-^ double-negative B cells, and CD27^high^CD38^high^ antibody-secreting cells was increased in patients with NMOSD. Tocilizumab treatment led to a significant shift of B cells to naïve B cells from memory B cells after 3 months. Three markers on B cells associated with T-cell activation (i.e., CD86 CD69, and HLA-DR) were downregulated after tocilizumab treatment. The frequencies of total Tfh and Tph cells were decreased, whereas that of follicular regulatory T cells tended to increase. Intrinsic increased PD-L1 and PD-L2 expression was characteristic of B cells in patients with NMOSD. Tocilizumab selectively restored PD-L1 on B-cell subsets. These results provided evidence that tocilizumab enhanced B- and T-cell homoeostasis by regulating B-cell differentiation and inhibiting lymphocyte activation in patients with NMOSD.

## Introduction

Neuromyelitis optica spectrum disorder (NMOSD) is an autoimmune inflammatory disease of the central nervous system involving pathogenic autoantibodies against aquaporin-4 (AQP4-IgG) (1, 2). Imbalances in B- and T-cell homoeostasis have been investigated. Among these, autoreactive B-cell subsets and loss of anergic maintenance may affect disease activity (3–5). The function of B cells is involved in antigen presentation, pro-inflammatory and anti-inflammatory cytokine production and immunoglobulin production. Circulating B cell subtypes include naïve B cells (CD19^+^CD27^-^IgD^+^) and 3 kinds of memory B cells, namely unswitched memory B cells (USW, CD19^+^CD27^+^IgD^+^IgM^+^), switched memory B (SWM, CD19^+^CD27^+^IgD^-^IgM^-^), double-negative (DN, CD19^+^CD27^-^IgD^-^) (3). Follicular T helper (Tfh) cells are critical in promoting B-cell autoimmunity and autoantibody production (6). In addition, peripheral helper T (Tph) cells was first defined with the markers of CD4^+^PD-1^high^CXCR5^-^. These cells expressed indispensable cytokines that enable B-cell help, including IL-21, CXCL13, ICOS, and MAF. Like Tfh cells, Tph cells can also induce plasma cell differentiation *in vitro* through IL-21 secretion and SLAMF5 interaction. Different from Tfh cells, Tph cells have unique expression of chemokine receptors (such as CCR2, CX3CR1, and CCR5) that direct migration to inflamed sites (7). However, the dynamics of Tph cells in NMOSD remain unclear. Specifically, AQP4-specific T cells are expanded in patients with NMOSD and exhibit pro-inflammatory Th17 polarization (8, 9).

Co-inhibitory signals characteristic of programmed cell death 1 (PD-1) are expressed on T cells as feedback for activated responses (10, 11). Engagement of PD-1 ligand 1 (PD-L1) and 2 (PD-L2) also plays important regulatory roles in immune responses (12). However, little is known about the kinetics of PD-1, PD-L1, and PD-L2 expression on B cells from patients with NMOSD.

Pleiotropic pro-inflammatory interleukin (IL)-6 drives disease activity and may contribute to abnormalities in B and T cells (13). Therapeutic agents targeting the IL-6 axis are effective in settings of acute and chronic inflammation (14). Recently, we reported a phase 2 randomized controlled trial showing that tocilizumab, a humanized anti-IL-6 receptor monoclonal antibody (mAb), significantly reduced the risk of relapse compared to azathioprine in NMOSD. Tocilizumab also decreases serum AQP4-IgG titers in patients with highly active NMOSD (15). However, the underlying effects of tocilizumab treatment on circulating B and T cells are unclear. Accordingly, in this study, we performed a detailed analysis of immunological phenotypes in B and T cells before and after tocilizumab treatment in patients with NMOSD.

## Material and Methods

### Recruitment and Enrollment of Patients and Controls

Patients with NMOSD were diagnosed according to 2015 International Panel for Neuromyelitis Optica Diagnosis criteria. Patients who received tocilizumab treatment at Tianjin Medical University General Hospital between November 2017 and May 2019 were enrolled. The inclusion criteria in this study were as follows: those who received routine infusion of tocilizumab at a dose of 8 mg/kg every 4 weeks for at least 3 months. At study entry, patients were permitted to continue the baseline treatment of corticosteroids. The exclusion criteria were as follows: 1) patients who were concomitantly treated with oral immunosuppressants, such as azathioprine, mycophenolate mofetil, and tacrolimus; and 2) patients who received rituximab treatment for less than 6 months from initial tocilizumab infusion. We also included 20 age- and sex-matched healthy controls (HCs) who had no malignancy or autoimmune disorder and did not receive immunosuppressive therapy. The Institutional Review Board of Tianjin Medical University General Hospital reviewed and approved this study. Each participant provided written informed consent.

### Flow Cytometry Analysis

All patients who received routine tocilizumab treatment were registered for immunophenotyping analysis. Peripheral blood was collected at baseline, 1 month, and 3 months after initiation of treatment. All fresh samples were immediately processed. Briefly, peripheral blood mononuclear cells (PBMCs) were prepared by density-gradient centrifugation, resuspended in phosphate-buffered saline (PBS)/3% human IgG (Bax International Inc., Vienna, Austria) to block Fc receptors and prevent nonspecific antibody binding, and then incubated for 15 min at 4°C in the dark. Subsequently, the cells were washed with PBS containing 1% bovine serum albumin. Background fluorescence was assessed using appropriate isotype- and fluorochrome-matched control mAbs.

To analyze the frequency of B-cell subpopulations, PBMCs were stained with allophycocyanin (APC) anti-human CD19 antibodies (clone HIB19), phycoerythrin (PE)/cyanine7 anti-human IgD antibodies (clone IA6-2), Alexa Fluor 488 anti-human CD38 antibodies (clone HIT2), Brilliant Violet 421 anti-human CD27 antibodies (clone M-T271), Alexa Fluor 488 anti-human HLA-DR antibodies (clone L243), APC anti-human CD86 antibodies (clone IT2.2), Brilliant Violet 421 anti-human CD69 antibodies (clone FN50), PE anti-human CD279 (PD-1) antibodies (clone EH12.2H7), APC anti-human CD274 (B7-H1, PD-L1) antibodies (clone 29E.2A3), and PE anti-human CD273 (B7-DC, PD-L2) antibodies (clone 24F.10C12). To characterize T-cell phenotypes, PBMCs were stained with fluorescein isothiocyanate anti-human CD4 antibodies (clone A161A1), Brilliant Violet 421 anti-human CD183 (CXCR3) antibodies (clone G025H7), PE/cyanine7 anti-human CD185 (CXCR5) antibodies (clone J252D4), APC/cyanine7 anti-human CD196 (CCR6) antibodies (clone G034E3), PE anti-human CD25 antibodies (clone BC96), and Brilliant Violet 421 anti-human CD127 (IL-7Rα) antibodies (clone A019D5). All the antibodies were purchased from Biolegend (San Diego, CA, USA).

Prior to the measurements, 1 μL of 300 nM 4′,6-diamidino-2-phenylindole (Invitrogen, Carlsbad, CA, USA) was added to exclude dead cells. The stained samples were assessed by flow cytometry using a FACS Aria III flow cytometer (BD Biosciences, San Jose, CA, USA). The results were analyzed using FlowJo software (version 10).

### Statistical Analysis

All statistical analyses were performed using GraphPad Prism v8 and R software. Continuous and ordinal variables are presented with medians (interquartile ranges [IQRs]) or means (standard deviations [SDs] or standard errors of the means [SEMs]). Shapiro-Wilk tests were used to determine whether the variables had a normal distribution. For comparisons between patients and HCs, unpaired, two-tailed Student’s *t* tests or Mann-Whitney *U* tests were used. Paired Student’s *t* tests (parametric) or Wilcoxon matched pairs tests (nonparametric) were used to compare baseline and post-treatment results in patients with NMOSD. For multiple comparisons, the Bonferroni-Dunn adjustment was used for analysis of repeated-measures. *P* values less than 0.05 were considered significant.

## Results

### Baseline Characteristics

The baseline characteristics of patients with NMOSD and HCs are described in **Table 1**. The median age of patients with NMOSD was 49.5 (IQR: 36.5–58.0) years. Additionally, 25 (96.3%) patients were women, and 24 (92.3%) patients were AQP4-IgG seropositive. The median duration of NMOSD was 2.59 (IQR: 0.63–4.10) years, and the median Expanded Disability Status Scale at baseline was 2.5 (IQR: 2.0–3.9). At baseline, 20 (76.9%) patients were being treated with oral corticosteroids (10mg/day, QD). They were permitted to receive corticosteroids for the first 12 weeks; thereafter, tocilizumab was used as monotherapy.

**Table 1:** Demographics of participants in the study.

	HCs (n=20)	NMOSD (n=26)
Female, n (%)	19 (95.0)	25 (96.3)
Age at baseline, median (IQR)	43.0 (39.3-51.8)	49.5 (36.5-58.0)
Age at onset, median (IQR)	—	45.5 (34.0-57.3)
Disease duration(y), median (IQR)	—	2.59 (0.63-4.10)
ARR at baseline, median (IQR)	—	0.28 (0.00-0.64)
EDSS score, median (IQR)	—	2.5 (2.0-3.9)
AQP4-ab positive, n (%)	—	24 (92.3)
Preventive medications at baseline, n (%)		
No treatment	—	6 (23.1)
oral corticosteroids		20 (76.9)

EDSS, Expanded Disability Status Scale; HC, healthy control; NMOSD, neuromyelitis optica spectrum disorders; IQR, interquartile range; Treatment was defined as use of high-dose intravenous steroids, plasma exchange (PE) or intravenous immunoglobulin (IVIG) at relapse stage; Values indicate median (interquartile range) or number (percent).

Relapse was defined as new onset of neurological symptoms or worsening of existing neurological symptoms with an objective change on neurological examination that persisted for more than 24 h, with signs and symptoms attributable solely to NMOSD, and preceded by at least 30 days of clinical stability. By the end of the study, three (11%) of 26 patients in the tocilizumab group had a relapse.

### Tocilizumab Treatment Restored the Distribution of B-Cell Subsets

**Figure 1:**
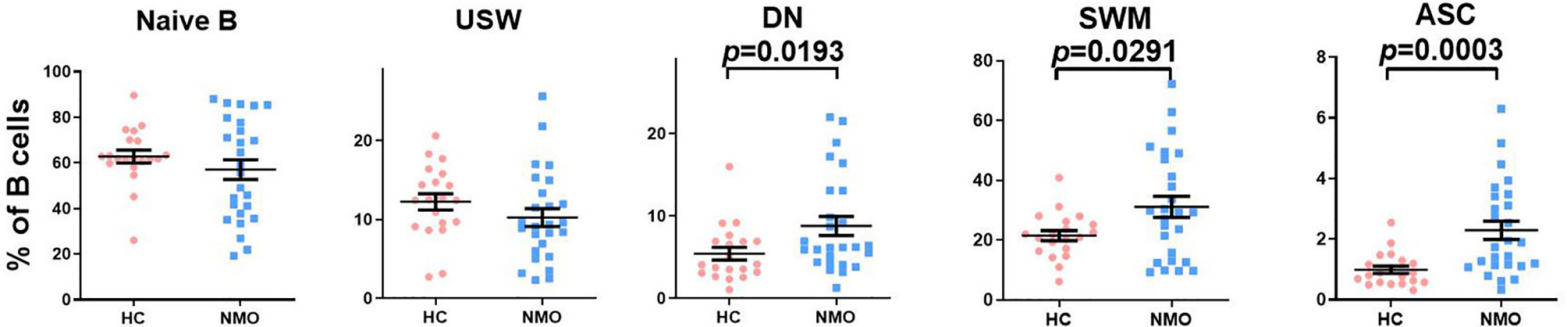
The comparison of B cell subsets distribution in healthy controls and patients with NMOSD. Proportions of CD27^-^IgD^+^ naïve B cell, CD27^+^IgD^+^ unswitched memory B (USW), CD27^-^IgD^-^ double negative B cell (DN), CD27^+^IgD^−^ switched memory B (SWM) cells and CD27^high^CD38^high^ autoantibodies secreted cells (ASC) among total B cells in patients with NMOSD and healthy controls (HC). Unpaired, two-tailed Student’s t tests were used. HC: n=20; NMOSD: n=26.

As shown in **Figure 1**, compared with HCs, patients with NMOSD exhibited significantly higher proportions of CD27^+^IgD^-^ switched memory (SWM) B cells (HC *versus* NMOSD:21.54% ± 1.69% *versus* 31.16% ± 3.53%, respectively; *P* = 0.0291), CD27^-^IgD^-^ double negative (DN) B cells (HC *versus* NMOSD: 5.41% ± 0.76% *versus* 8.80% ± 1.16%, respectively; *P* = 0.0193), and CD27^high^CD38^high^ antibody-secreting cells (ASCs) (HC *versus* NMOSD: 0.98% ± 0.12% *versus* 2.29% ± 0.30%, respectively; *P* = 0.0003), but statistical analysis showed no significant differences between HCs and NMOSD patients in the proportion of CD27^-^IgD^+^ naïve B cells (HC *versus* NMOSD: 62.80% ± 2.83% *versus* 57.05% ± 4.35%, respectively; *P* = 0.2742) and CD27^+^IgD^+^ unswitched memory (USM) B cells (HC *versus* NMOSD: 12.23% ± 1.03% *versus* 10.24% ± 1.14%, respectively; *P* = 0.2015). That is to say, the distribution of B-cell subsets was different from that of HCs, with an elevated proportion of DN, SWM cells, and ASCs.

**Figure 2:**
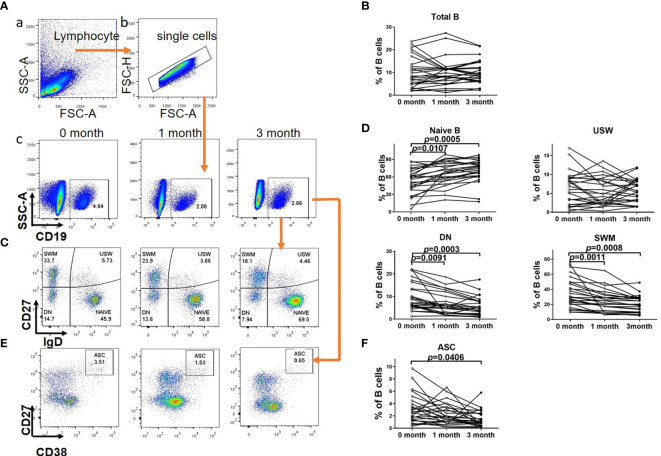
B cell subsets distribution in NMOSD patients before and after tocilizumab. **(A)** Gating strategy for B subsets. (a) Sideward scatter-A (SSC-A) and Forward scatter-A (FSC-A) were used for lymphocytes gating. (b) FSC-H and FSC-A were used to gate single cells. (c) B cells was charactered by CD19^+^. **(B)** Quantitative analysis of proportion of total B cells. The effect of tocilizumab treatment on these circulating B cell subsets was also assessed in NMOSD patients at baseline and after 1 month/3 months of treatment. **(C, E)** Representative flow cytometry of CD19^+^CD27^–^IgD^+^ (naive), CD19^+^CD27^–^IgD^–^ (double-negative, DN), CD19^+^CD27^+^IgD^+^ (unswitched memory, USW), CD19^+^CD27^+^IgD^–^ (switched memory, SWM) and CD19^+^CD27^high^ CD38^high^ ASC (Autoantibodies secreted cells) from NMOSD patients before and after tocilizumab. **(D, F)** Quantitative analysis of proportion of B cell subsets and ASC. Significance was tested by repeated measures of analysis of variance and the Bonferroni–Dunn adjustment for multiple comparisons. Paired, two-tailed Student’s *t* tests (if the distribution is normal) or Wilcoxon matched pairs tests (If it does not conform to the normal distribution) were used to compare baseline and post-treatment results, n=26.

There were no significant differences in the proportions of total B cells in lymphocyte cells at 1 or 3 months after tocilizumab treatment compared with those at baseline (1 month *versus* 0 month: 9.98% ± 1.19% *versus* 10.19% ± 1.32%, respectively, *P* = 0.8027; 3 months *versus* 0 month: 10.14% ± 1.03% *versus* 10.19% ± 1.32%, respectively, *P* = 0.9332) (**Figures 2A, B**). When come to the B cell subtypes, it was found that the frequencies of DN (1 month *versus* 0 month: 6.86% ± 0.93% *versus* 9.86% ± 1.21%, respectively, *P* = 0.0091) and SWM B cells (1 month *versus* 0 month: 24.58% ± 2.83% *versus* 33.74% ± 3.52%, respectively, *P* = 0.0011) were significantly decreased compared with those at the baseline after 1 month of tocilizumab treatment in patients with NMOSD. In contrast, the frequency of naïve B cells was significantly increased (1 month *versus* 0 month: 65.91% ± 3.92% *versus* 52.54% ± 4.25%, *P* = 0.0107). We did not find a significant decrease in the frequencies of ASCs (1 month *versus* 0 month: 2.28% ± 0.33% *versus* 3.06% ± 0.46%, *P* = 0.0863) or unswitched memory (USM) B cells (1 month *versus* 0 month: 5.38% ± 0.72% *versus* 6.32% ± 0.86%, *P* = 0.1147). After 3 months of treatment, tocilizumab led to a significant shift to a more naïve B-cell phenotype from mature memory B cells, as demonstrated by an increase in naïve B cell numbers (3 month *versus* 0 month: 70.43% ± 3.76% *versus* 52.54% ± 4.25%, *P* = 0.0005) and decreases in SWM (3 month *versus* 0 month: 20.71% ± 2.10% *versus* 33.74% ± 3.52%, *P* = 0.0008) and DN B cell numbers (3 month *versus* 0 month: 5.62% ± 0.80% *versus* 9.86% ± 1.21%, *P* = 0.0003) compared with those at baseline, After 3 months of treatment, the proportion of abnormally elevated ASCs also decreased (3 month *versus* 0 month:1.47% ± 0.26% *versus* 3.06% ± 0.46%, *P* = 0.0406) (**Figures 2C-F**). Although not significant, tocilizumab tended to decrease the proportions of USM B cells at 3 months (3 month *versus* 0 month:5.90% ± 0.60% *versus* 6.32% ± 0.86%, *P* = 0.0791). We did not find any significant differences between the proportions of naïve B cells (1 month *versus* 3 month: 65.91% ± 3.92% *versus* 70.43% ± 3.76%, *P*=0.0943), DN B cells(1 month *versus* 3 month: 6.86% ± 0.93% *versus* 5.62% ± 0.80%, *P*=0.0610), SWM B cells (1 month *versus* 3 month: 24.58% ± 2.83% *versus* 20.71% ± 2.10%, *P*=0.1023), USM B cells(1 month *versus* 3 month: 5.38% ± 0.72% *versus* 5.90% ± 0.60%, *P*=0.4315) and ASC (1 month *versus* 3 month: 2.28% ± 0.33% *versus* 1.47% ± 0.26%, *P*=0.0989) at 1 month of tocilizumab treatment and those at 3 months.

In a word, tocilizumab treatment restored the distribution of B-cell subsets, to be detailed, the elevated proportion of DN, SWM cells and ASCs was partial recovered, otherwise the proportion of naïve B cells increased.

### Tfh, Peripheral T Helper, and Follicular Regulatory T Cells Were Differentially Affected in Tocilizumab-Treated Patients With NMOSD

**Figure 3:**
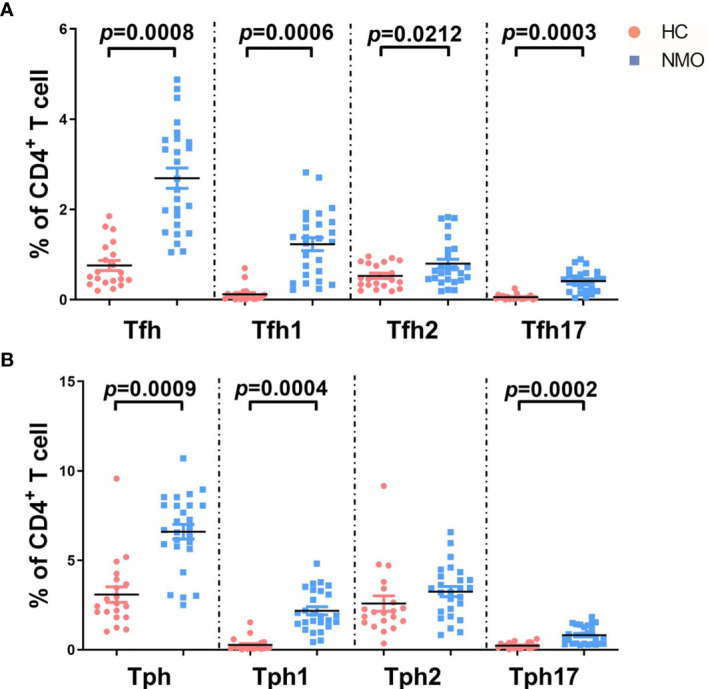
The comparison of T cell subsets distribution in healthy controls and patients with NMOSD. **(A)** Proportions of T follicular helper (Tfh: CD4^+^CXCR5^+^PD-1^high^) and subsets among total CD4^+^ cells in patients with NMOSD and healthy controls (HC); **(B)** Proportions of peripheral T helper (Tph: CD4^+^CXCR5^-^PD-1^high^) and subsets. Tfh1 (CXCR3^+^CCR6^–^), Tfh2 (CXCR3^–^CCR6^–^), and Tfh17 (CXCR3^–^CCR6^+^), Tph1 (CXCR3^+^CCR6^–^), Tph2 (CXCR3^–^CCR6^–^), and Tph17 (CXCR3^–^CCR6^+^). Unpaired, two-tailed Student’s t tests were used. HC: n=20; NMOSD: n=26.

First, we examined the proportions of Tph (CD4^+^CXCR5^-^PD-1^high^) and Tfh (CD4^+^CXCR5^+^PD-1^high^) cells in the peripheral blood of patients with NMOSD at baseline. We found that the proportions of both phenotypes were significantly higher in the patients with NMOSD than those in HCs (Tfh cells: 0.76% ± 0.11% *versus* 2.69% ± 0.23%, respectively, *P*= 0.0008; Tph cells: 3.08% ± 0.45% *versus* 6.59% ± 0.43%, respectively, *P* = 0.0009). Moreover, proportions of the Tfh cell subsets Tfh1 (0.12% ± 0.04% *versus* 1.23% ± 0.14%, respectively; *P* = 0.0006), Tfh17 (0.05% ± 0.01% *versus* 0.41% ± 0.05%, respectively; *P* = 0.0003), and Tfh2 (0.52% ± 0.06% *versus* 0.79% ± 0.10%, respectively; *P* = 0.0212) were significantly increased in patients with NMOSD. Additionally, proportions of the Tph cell subsets Tph1 (0.27% ± 0.08% *versus* 2.12% ± 0.22%, respectively; *P* = 0.0004) and Tph17 (0.22% ± 0.03% *versus* 0.80% ± 0.12%, respectively; *P* = 0.0002) were increased, whereas the proportion of Tph2 cells (2.58% ± 0.43% *versus* 3.25% ± 0.29%, respectively; *P* = 0.1836) was not significantly decreased (**Figures 3A, B**).

**Figure 4:**
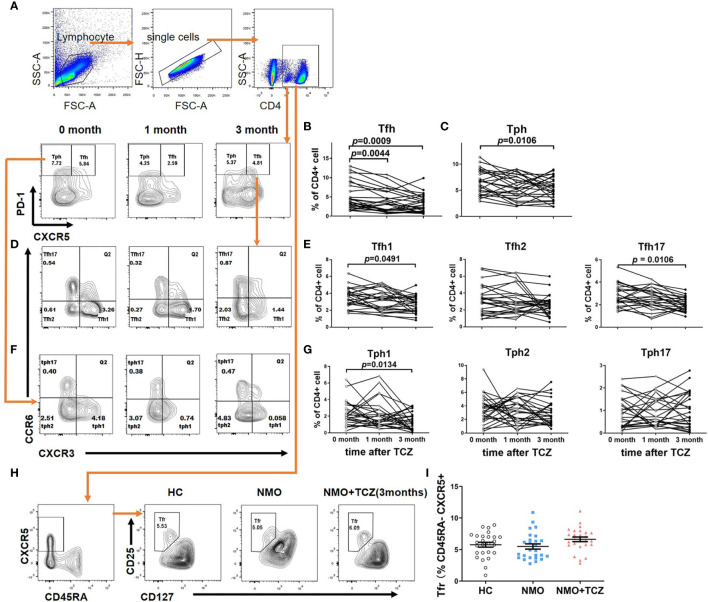
T cells subsets distribution in NMOSD patients before and after tocilizumab. **(A, D, F)** showed gating strategy for subsets of T Follicular Helper Cell (Tfh) & peripheral T helper cells (Tph) and representative flow cytometry plot at baseline and after treatment for 1month or 3 months. **(B, C, E, G)** Quantitative analysis of proportion of total Tfh and its subsets: Tfh1 (CD4^+^CXCR5^+^PD-1^high^CXCR3^+^CCR6^–^), Tfh2 (CD4^+^CXCR5^+^PD-1^high^CXCR3^–^CCR6^–^) and Tfh17 (CD4^+^CXCR5^+^PD-1^high^CXCR3^–^CCR6^+^), total Tph and its subsets: Tph1 (CD4^+^CXCR5^-^PD-1^high^CXCR3^+^CCR6^–^), Tph2 (CD4^+^CXCR5^-^PD-1^high^CXCR3^–^CCR6^–^), and Tph17 (CD4^+^CXCR5^-^PD-1^high^CXCR3^–^CCR6^+^) at baseline and after treatment for 1month or 3 months (n=19). **(H)** Gating strategy for T follicular Regulatory cells (Tfr) and flow cytometry plot of a control and a representative NMOSD patient (before and after tocilizumab 3 months). **(I)** Quantitative analysis of proportion of Tfr in healthy controls and NMOSD patient before and after tocilizumab 3 months (n=26). Differences were considered statistically significant for *P*<0.05. Paired, two-tailed Student’s *t* tests (if the distribution is normal) or Wilcoxon matched pairs tests (if it does not conform to the normal distribution) were used to compare baseline and post-treatment results, n=26.

No significant differences were found in the proportions of CD4^+^CD45RA^-^CXCR5^+^CD25^+^CD127^-^ Tfr cells in patients with NMOSD compared to those in HCs (5.49% ± 0.41% *versus* 5.77% ± 0.39%, respectively; *P* = 0.6273; **Figure 4I**).

Next, we assessed the effects of tocilizumab on Tfh, Tph, and Tfr cells. After tocilizumab treatment, there were significantly lower proportions of total Tfh cells at 1 month (0 month *versus* 1 month: 5.10% ± 0.70% *versus* 3.86% ± 0.57%, *P* = 0.0044) and 3 months (0 month *versus* 3 months: 5.10% ± 0.70% *versus* 3.18% ± 0.45%, *P* = 0.0009) compared to those before treatment (**Figures 4A, B**). Tocilizumab treatment tended to reduce the number of total Tph cells at 1 month (0 month *versus* 1 month: 6.61% ± 0.46% *versus* 5.61% ± 0.45%, *P* = 0.1884), and a significant decrease in the number of Tph cells was observed after 3 months (0 month *versus* 3 months: 6.61% ± 0.46% *versus* 5.38% ± 0.42%, *P* = 0.0106; **Figure 4C**). In addition, among the Tfh cell subsets, we observed a tendency for reduction in the proportion of Tfh1 cells (0 month *versus* 1 month: 3.54% ± 0.22% *versus* 3.15% ± 0.24%, *P* = 0.0929) and Tfh17 cells (0 month *versus* 1 month: 2.83% ± 0.19% *versus* 2.56% ± 0.18%, *P* = 0.1049) at 1 month, and a significant decrease in the number of Tfh1 cells (0 month *versus* 3 month: 3.54% ± 0.22% *versus* 2.68% ± 0.20%, *P* = 0.0491) and Tfh17 cells (0 month *versus* 3 months: 2.83% ± 0.19% *versus* 2.03% ± 0.12%, *P* = 0.0106) at 3 months. There was no significant decrease in the proportion of Tph1 at 1 month of tocilizumab treatment (0 month *versus* 1 month: 2.34% ± 0.28% *versus* 2.16% ± 0.33%, *P* = 0.6356) until 3 months (0 month *versus* 3 months: 2.34% ± 0.28% *versus* 1.35% ± 0.21%, *P* = 0.0134). We did not find any significant changes in the frequencies of Tfh2 cells, Tph2 cells or Tph17 cell subsets after tocilizumab treatment (**Figures 4D–G**). The proportion of Tfr cells tended to increase; however, the difference was not statistically significant (0 month *versus* 3 months:5.77% ± 0.39% *versus* 6.63% ± 0.37%, respectively; *P* = 0.1143; **Figures 4H, I**).

To sum up, the frequencies of total Tfh and Tph cells were decreased, whereas that of follicular regulatory T cells tended to increase. The subtypes of Tfh1, Tfh17 and Tph1 cells decreased significantly.

### Tocilizumab Inhibited the Activation of B Cells in Patients With NMOSD

**Figure 5:**
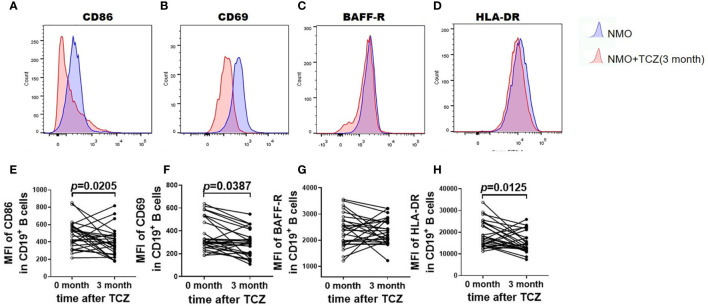
Tocilizumab alters the expression level of activation marker. **(A–D)** was the representative flow cytometric histogram after surface staining activation marker on total B cells (CD19^+^) from NMOSD patients before and after tocilizumab treatment. **(E–H)** was the quantitative analysis of the mean fluorescence intensity (MFI) of CD86, CD69, BAFF-R, HLA-DR in CD19^+^cells before and after tocilizumab treatment. Difference was considered statistically significant for *P* < 0.05. Paired, two-tailed Student’s *t* tests were used, n=26.

Next, we studied the expression of the costimulatory molecules CD86 and CD69 as markers of activated B cells that modulate T-cell signaling. No significant changes in the mean fluorescence intensity (MFI) of CD86 or CD69 were observed in CD19^+^ B cells after tocilizumab treatment for 1 month (not shown); however, the MFI of CD86 was decreased after 3 months (488.2 ± 30.47 *versus* 392.3 ± 32.62, respectively; *P* = 0.0205) and that of CD69 decreased at 3 months (366.5 ± 25.87 *versus* 291.6 ± 22.50, respectively; *P* = 0.0387; **Figures 5A–F**). There were no significant differences in the expression of BAFF-R (2384 ± 124.9 *versus* 2336 ± 96.75, respectively; *P* = 0.7620) before and after treatment with tocilizumab (**Figures 5G, H**). In contrast, HLA-DR expression decreased significantly after 3 months of tocilizumab treatment (19017 ± 1174 *versus* 14550 ± 1033, respectively; *P* = 0.0125). In short, three markers on B cells associated with T-cell activation (i.e., CD86 CD69, and HLA-DR) were downregulated after tocilizumab treatment.

### Effects of Tocilizumab Treatment on the Expression of PD-1 and Its Ligands in Peripheral B Cell Subsets

**Figure 6:**
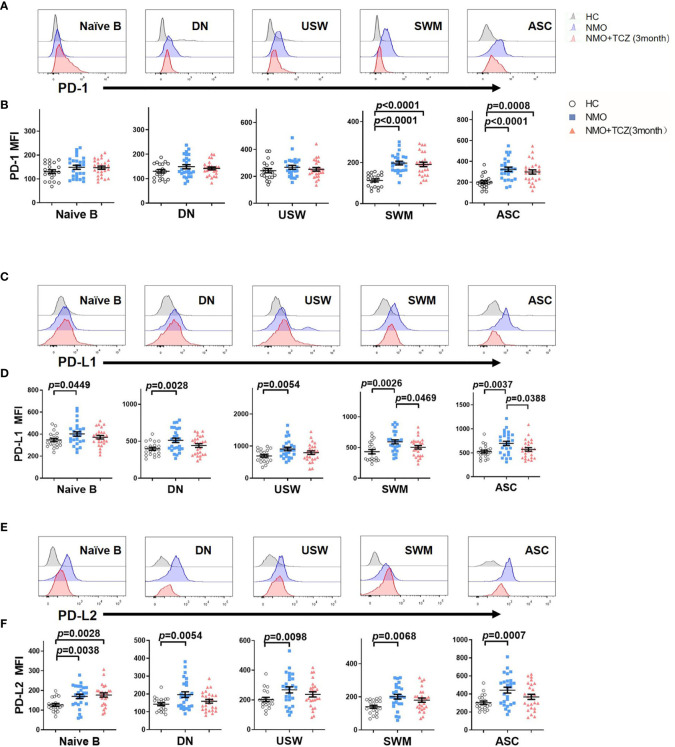
The expression of PD-1 and its corresponding ligands PD-L1 and PD-L2 on B cells subsets from healthy control (HC) and NMOSD patients before and after treatment. **(A, C, E)** Representative histograms of PD-1, PD-L1, PD-L2 in several B cells subsets from a healthy control and NMOSD patients before and after treatment. **(B, D, F)** was the quantitative analysis of mean fluorescence intensity (MFI) of PD-1, PD-L1&PD-L2 in several B cells subsets. Unpaired, two-tailed Student’s *t* tests (if the distribution is normal) or Mann-Whitney *U* tests (if it does not conform to the normal distribution) were used, n=26.

First, we compared the expression of PD-1 and its ligands PD-L1 and PD-L2 on B-cell subsets in HCs and patients with NMOSD. SWM B cells and ASCs exhibited significantly higher PD-1 expression in patients with NMOSD than that in HCs (NMOSD *versus* HCs: 197.7 ± 8.93 *versus* 113.2 ± 6.64, respectively; *P*<0.0001 and 323.5 ± 20.07 *versus* 200.1 ± 14.50 respectively; *P* < 0.0001). There were no significant differences in the expression of PD-1 on naïve B cells (NMOSD *versus* HCs): (148.5 ± 8.43 *versus* 130.5 ± 8.07, respectively; *P*=0.1378), DN B cells (147.9 ± 8.52 *versus* 130.3 ± 7.01, respectively; *P*=0.1201), and USW B cells (266.9 ± 14.47 *versus* 241.9 ± 15.80, respectively; *P*=0.2318) (**Figures 6A, B**). Intriguingly, we found that all B-cell subsets expressed higher levels of PD-L1 and PD-L2 in patients with NMOSD compared with HCs, to be detailed, the comparison of PD-L1 and PD-L2 levels (HCs *versus* NMOSD) on B cell subsets is that: naïve B (PD-L1: 346.8 ± 15.41 *versus* 401.6 ± 19.99, *P*=0.0449; PD-L2:126.7 ± 7.68 *versus* 170.0 ± 10.93, respectively; *P*=0.0038); DN B cells (PD-L1: 397.2 ± 20.57 *versus* 512.3 ± 29.88, *P*=0.0028; PD-L2:142.4 ± 8.59 *versus* 195.9 ± 15.94, respectively; *P*=0.0054); USW B cells (PD-L1: 692.6 ± 43.27 *versus* 908.7 ± 55.48, *P*=0.0054; PD-L2:203.1 ± 14.41 *versus* 267.9 ± 20.82, respectively; *P*=0.0098); SWM B cells (PD-L1: 432.5 ± 35.63 *versus* 592.9 ± 32.93, *P*=0.0026; PD-L2:140.0 ± 9.06 *versus* 200.8 ± 14.14, respectively; *P*=0.0068) and ASCs (PD-L1: 526.6 ± 30.41 *versus* 695.0 ± 45.53, *P*=0.0037; PD-L2:302.7 ± 18.76 *versus* 441.6 ± 32.68, respectively; *P*=0.0007) (**Figures 6C–F**).

Next, we compared the expression of PD-1 and its ligands on B-cell subsets in the patients with NMOSD after 3 months of treatment with those in HCs. SWM B cells and ASCs exhibited significantly higher PD-1 expression in patients with NMOSD after treatment than that in HCs (191.0 ± 10.96 *versus* 113.2 ± 6.64, respectively; *P*<0.0001 and 297.6 ± 20.43 *versus* 200.1 ± 14.50 respectively; *P* = 0.0008). There were no significant differences in the expression of PD-1 on naïve B cells (HCs *versus* NMOSD+TCZ: 130.5 ± 8.07 *versus* 147.2 ± 6.71, *P*=0.1431), DN B cells (147.9 ± 8.52 *versus* 130.3 ± 7.01, respectively; *P*=0.2120), and USW B cells (266.9 ± 14.47 *versus* 241.9 ± 15.80, respectively; *P*=0.4582). Statistically, tocilizumab did not significantly affect the expression of PD-L1 or PD-L2 on all B-cell subsets compared with the baseline except in naïve B cells (PD-L2:126.7 ± 7.68 *versus* 177.1 ± 10.55, respectively; *P*=0.0028), although a decreasing tendency was observed in several B-cell subsets. To be detailed, the comparison of PD-L1 and PD-L2 levels (HCs *versus* NMOSD+TCZ) on B cell subsets is that: naïve B (PD-L1: 346.8 ± 15.41 *versus* 372.0 ± 15.39, *P*=0.2630); DN B cells (PD-L1: 397.2 ± 20.57 *versus* 441.1 ± 24.45, *P*=0.1936; PD-L2:142.4 ± 8.59 *versus* 158.8 ± 10.47, respectively; *P*=0.2523); USW B cells (PD-L1: 692.6 ± 43.27 *versus* 794.8 ± 54.56, *P*=0.1680; PD-L2: 203.1 ± 14.41 *versus* 237.4 ± 17.43, respectively; *P*=0.1150); SWM B cells (PD-L1: 432.5 ± 35.63 *versus* 503.7 ± 28.82, *P*=0.1026; PD-L2:140.0 ± 9.06 *versus* 179.9 ± 12.96, respectively; *P*=0.0634) and ASCs (PD-L1: 526.6 ± 30.41 *versus* 571.4 ± 36.32, *P*=0.3676; PD-L2: 302.7 ± 18.76 *versus* 367.1 ± 28.63, respectively; *P*=0.0667).

Finally, we compared the expression of PD-1 and its ligands on B-cell subsets in the patients with NMOSD before and after 3 months of treatment. Statistical analysis shows that the expression of PD-L1 on SWM cells and ASCs after treatment was significantly lower than those before treatment (592.9 ± 32.93 *versus* 503.7 ± 28.82, *P*=0.0469 and 695.0 ± 45.53 *versus* 571.4 ± 36.32, *P*=0.0388 respectively) (**Figure 6B**). Additionally, there was no significant differences in the expression of PD-1 and its ligands on B-cell subsets (**Figures 6D, F**).

As a whole, tocilizumab-treated patients showed little changes in PD-1 expression among all B-cell subsets compared with the baseline. By contrast, treatment with tocilizumab does have a tendency to reduce PD-L1 and PD-L2 expression on B-cell subsets. For instance, the expression of PD-L1 on SWM cells and ASCs after treatment was significantly lower than those before treatment (**Figures 6D, F**).

## Discussion

The differential effects of disease-modifying drugs (DMDs) on peripheral blood B-cell subsets and differentiation-related Th cells have been investigated in multiple sclerosis (16–18). However, few studies have reported the effects of DMDs in NMOSD.

In this study, we found that *in vivo* blockade of the IL-6 receptor decreased lymphocyte activation, altered B- and T-cell homoeostasis in patients with NMOSD, and promoted the normalization of abnormal B- and T-cell subsets observed at baseline. Specifically, tocilizumab treatment led to an increase in the number of naïve B cells and decreases in the number of memory B cells and ASC B cells. Similarly, the frequencies and absolute numbers of abnormally reduced Tfh cells increased.

Decreases in the number of memory B cells were consistent with those reported with tocilizumab in patients with rheumatoid arthritis in a previous study (19). Unlike in rheumatoid arthritis, however, we also observed a significant decrease in the number of ASCs, probably because of the more pronounced abnormalities of these cells in patients with NMOSD. Naïve B cells are also dysregulated in patients with NMOSD, as reflected by the lower frequency of naïve B cells. Remarkably, the frequency of naïve B cells was normalized in response to tocilizumab, and the changes in naïve B cells were attributed to the recovery of the reduced number of memory B cells, ASCs, and DN B cells and, to a lesser degree, to a decline in the number of USW B cells. Second, it is known that IL-6 plays an important role in the terminal differentiation of B cells. Thus, IL-6 receptor blockade may lead to a failure of differentiation from naïve B cells to memory B cells.

Double-negative B cells are thought to be mature antigen-experienced B cells with developmental marker expression profiles similar to those of post-switch memory B cells (20). The proportion of DN B cells is increased in patients with multiple sclerosis, resulting in increased production of pro-inflammatory and cytotoxic cytokines following ex-vivo stimulation (21, 22). Consistent with a previous study (23), we also observed expanded DN B cells in patients with NMOSD, suggesting the possible involvement of DN B cells in the pathogenesis of NMOSD (19, 24). In this study, tocilizumab treatment effectively reduced the numbers of DN B cells and may facilitate the inhibition of inflammation in NMOSD. DN B cells were further divided into two subgroups in a previous study on systemic lupus erythematosus (SLE), DN1 cells (CXCR5^+^ CD19^+^ IgD^-^CD27^-^) and DN2 cells (CXCR5^-^ CD19^+^IgD^-^ CD27^-^). DN2 cells express T-bet and CD11c, and their activation is mediated by hyper-responsiveness to TLR7 and leads to the generation of autoreactive ASCs (25). The deeper characterization of these cells, *via* immunophenotyping, has the potential to identify novel SLE phenotypes that associate with disease-activity (26). Moreover, the frequency of DN B cells in rheumatic arthritis is inversely correlated to a subsequent good response (27). Additional larger scale studies are needed to determine whether DN B cells may serve as predictors of treatment response to tocilizumab.

In this research, blockade of the IL-6 receptor restores abnormally elevated SWM B cells and ASCs seen at baseline. The decrease of SWM B cells and ASCs may explain the reduction of the pathogenic AQP4-IgG titers found in our previous clinical trial (15), which showed that among AQP4-IgG positive patients, tocilizumab decreased sera AQP4-IgG titers significantly by 50% at the end of the study compared with those at baseline.

In NMOSD, the balance in Tfh cells and related subsets is impaired, making these cells a potential therapeutic target. Consistent with previous studies, we found an upregulation of Tfh cells, particularly Tfh1 cells, in NMOSD patients compared with HCs (6, 28, 29), while 3 months of treatment with tocilizumab reduced the frequency of Tfh cells, and we found that Tfh cell differentiation was associated with IL-6 and plasmablast formation. In line with our findings, specific targeting of IL-6 using tocilizumab therapy in patients with rheumatoid arthritis can significantly reduce circulating Tfh cell numbers, which are correlated with reduced plasmablast formation (30). In this study, we observed similar results in tocilizumab-treated patients with NMOSD, indicating the therapeutic efficacy of tocilizumab in these patients.

Tph cells may play a role in promoting B-cell responses and antibody production within pathologically inflamed non-lymphoid tissues. The possible mechanism lies in that CXCL13 and IL-21 produced by Tph cells may recruit both Tfh and B cells and thus promote local autoantibody production and perhaps modulate other B cell functions such as cytokine production (31). We found that Tph cells were increased in patients with NMOSD. Tocilizumab treatment resulted in reduction of the number of peripheral blood Tph cells. Further research is needed to investigate if this can offer a potential strategy for therapeutic targeting of tissue T cell-B cell interactions.

The role of IL-6 in autoimmune diseases helps to understand the mechanism of the effect of tocilizumab on B- and T-cell subsets. For B cells, IL-6 can promote the terminal differentiation of B cells (32) and contribute to survival of plasma cells (33). IL-6 receptor blockade by tocilizumab is expected to inhibit the differentiation of B cells and survival of plasma cells, as both of them express the IL-6 receptor. For T cells, IL-6 is known to promote early Tfh cell differentiation by transiently inducing B cell lymphoma 6 (BCL-6) expression in CD4^+^ T cells (34). Further, Tfh cells can promote germinal center formation through the production of IL-21, which sustains BCL-6 expression on B cells and promotes B cell activation, class-switch recombination, and plasma cell differentiation (35). Tph cell is a recently discovered Th cell subset, which can also promote B cell differentiation by secreting IL-21 (36). In general, pro-inflammatory IL-6 promotes the formation of germinal center B cells and Tfh/Tph cells in cooperation with IL-21.

The PD-1 pathway is one of the most important immune checkpoints and is indispensable for maintaining the homeostasis and tolerance of the immune system. The expression of PD-1 family members on B cells in NMOSD has not yet been investigated in detail. Herein, we found that PD-1, PD-L1, and PD-L2 expression was increased on ASCs and memory B cells in NMOSD patient compared to that in healthy controls, consistent with previous studies in systemic lupus erythematosus (37). Because all B cells are pro-inflammatory in NMOSD, upregulation of PD-1 and its ligands on B cells may on account of cellular activation. Additionally, PD-L1-deficient B cells in an experimental autoimmune encephalomyelitis mouse model exhibit aggravated autoimmunity (38), suggesting that the PD-1 pathway may have roles in the ablation of autoimmunity. In our cohort, tocilizumab treatment selectively downregulated PD-L1 expression on ASCs and memory B cells in patients with NMOSD; this treatment could block or control autoimmune responses and help restore homeostasis. Although B cells expressing PD-1 may diminish after anti-CD19 or anti-CD20 mAb treatment, the effects of tocilizumab on PD-1-related co-inhibitory signaling are persistent. This indicates that the mechanisms of tocilizumab differ from those of rituximab or inebilizumab regarding inhibition of disease activity. Little is known about the expression and kinetics of PD-1, PD-L1, and PD-L2 by B lymphocytes from NMOSD patients. Not only B cells that express PD-L1 and PD-L2 and T cells expressing PD-1 interact *via* this pathway, but also B cell-expressed PD-1, that could mediate B-B or B-T interactions. We postulate that the PD-1 system on B cells acts as a feedback of neuroinflammation in NMOSD. The elevated PD-1 system on B cells may play a protective role in controlling autoimmunity in active NMOSD. When autoimmune inflammation is suppressed by tocilizumab treatment, the PD-1 system tends to restore.

This study has several limitations. First, the follow-up period was relatively short, and the long-term effects on B-cell subsets after tocilizumab treatment were not evaluated. Second, the sample size of patients with tocilizumab treatment was extremely small. Additionally, 3 patients in this study experienced attacks during follow-ups, but the reason is not still clear. High-throughput single-cell sequencing may be warranted to explore heterogeneity of cellular immune function in patients who didn’t respond well to tocilizumab. Finally, it is a limitation that the mechanisms of the effects of tocilizumab on Tfh/Tph cell inhibition are not investigated in our study. Further evidence will be warranted on the mechanistic study.

## Conclusions

In summary, we showed that tocilizumab inhibited the activation of mature B cells and Tfh cells but maintained co-inhibitory PD-1 expression on B cells in the peripheral blood of patients with NMOSD. These findings provide important insights into the effects of tocilizumab on the immune mechanism of NMOSD.

## Data Availability Statement

The raw data supporting the conclusions of this article will be made available by the authors, without undue reservation.

## Ethics Statement

The studies involving human participants were reviewed and approved by The Institutional Review Board of Tianjin Medical University General Hospital. The patients/participants provided their written informed consent to participate in this study.

## Author Contributions

CZ and YL designed the study, collected, analyzed and interpreted the data and drafted and revised the manuscript. YL, PZ, and CD performed the research. YL and HZ did statistical analysis. T-XZ, MY, ZH, DJ, and GY collected and analyzed the data. F-DS revised the manuscript critically for intellectual content. All authors contributed to the article and approved the submitted version.

## Funding

The study was supported by grants from the National Natural Science Foundation of China (81601019, 81601039, 82071389) and the Natural Science Foundation of Tianjin Province (20JCJQJC00280, 18JCQNJC13200, 20JCQNJC00460).

## Conflict of Interest

The authors declare that the research was conducted in the absence of any commercial or financial relationships that could be construed as a potential conflict of interest.

## Publisher’s Note

All claims expressed in this article are solely those of the authors and do not necessarily represent those of their affiliated organizations, or those of the publisher, the editors and the reviewers. Any product that may be evaluated in this article, or claim that may be made by its manufacturer, is not guaranteed or endorsed by the publisher.

## Abbreviations

AQP4, aquaporin-4; ASCs, antibody-secreting cells; DN B cells, double negative B cells; HCs, healthy controls; MFI, mean fluorescence intensity; NMOSD, Neuromyelitis optica spectrum disorder; PBMCs, peripheral blood mononuclear cells; PD-1, programmed cell death 1; PD-L, programmed cell death 1 ligand; SWM B cells, switched memory B cells; Tfh cells, follicular T helper cells; Tfr cells, follicular regulatory T cells; Tph cells, peripheral T helper cells; USM B cells, unswitched memory B cells.

## References

[1] Wingerchuk D Lennon V Lucchinetti C Pittock S Weinshenker B. The Spectrum of Neuromyelitis Optica. Lancet Neurol (2007) 6:805–15. 10.1016/S1474-4422(07)70216-8 17706564

[2] Wingerchuk D Banwell B Bennett J Cabre P Carroll W Chitnis T. International Consensus Diagnostic Criteria for Neuromyelitis Optica Spectrum Disorders. Neurology (2015) 85:177–89. 10.1212/WNL.0000000000001729 PMC4515040 26092914

[3] Bennett J O’Connor K Bar-Or A Zamvil S Hemmer B Tedder T. B Lymphocytes in Neuromyelitis Optica. Neurol Neuroimmunol Neuroinflamm (2015) 2:e104. 10.1212/NXI.0000000000000104 25977932 PMC4426682

[4] Chihara N Aranami T Sato W Miyazaki Y Miyake S Okamoto T. Interleukin 6 Signaling Promotes Anti-Aquaporin 4 Autoantibody Production From Plasmablasts in Neuromyelitis Optica. Proc Natl Acad Sci USA (2011) 108:3701–6. 10.1073/pnas.1017385108 PMC3048150 21321193

[5] Wilson R Makuch M Kienzler AK Varley J Taylor J Woodhall M. Condition-Dependent Generation of Aquaporin-4 Antibodies From Circulating B Cells in Neuromyelitis Optica. Brain (2018) 141:1063–74. 10.1093/brain/awy010 PMC5889028 29447335

[6] Li Y Zhang F Qi Y Chang G Fu Y Su L. Association of Circulating Follicular Helper T Cells With Disease Course of NMO Spectrum Disorders. J Neuroimmunol (2015) 278:239–46. 10.1016/j.jneuroim.2014.11.011 25468778

[7] Rao DA Gurish MF Marshall JL Slowikowski K Fonseka CY Liu Y. Pathologically Expanded Peripheral T Helper Cell Subset Drives B Cells in Rheumatoid Arthritis. Nature (2017) 542:110–4. 10.1038/nature20810 PMC5349321 28150777

[8] Agasing A Wu Q Khatri B Borisow N Ruprecht K Brandt A. Transcriptomics and Proteomics Reveal a Cooperation Between Interferon and T-Helper 17 Cells in Neuromyelitis Optica. Nat Commun (2020) 11:2856. 10.1038/s41467-020-16625-7 32503977 PMC7275086

[9] Varrin-Doyer M Spencer C Schulze-Topphoff U Nelson P Stroud R Cree B. Aquaporin 4-Specific T Cells in Neuromyelitis Optica Exhibit a Th17 Bias and Recognize Clostridium ABC Transporter. Ann Neurol (2012) 72:53–64. 10.1002/ana.23651 22807325 PMC3405197

[10] Shi J Hou S Fang Q Liu X Liu X Qi H. PD-1 Controls Follicular T Helper Cell Positioning and Function. Immunity (2018) 49:264–74.e264. 10.1016/j.immuni.2018.06.012 30076099 PMC6104813

[11] Salama A Chitnis T Imitola J Ansari M Akiba H Tushima F. Critical Role of the Programmed Death-1 (PD-1) Pathway in Regulation of Experimental Autoimmune Encephalomyelitis. J Exp Med (2003) 198:71–8. 10.1084/jem.20022119 PMC2196082 12847138

[12] Khan A Hams E Floudas A Sparwasser T Weaver C Fallon P. PD-L1hi B Cells Are Critical Regulators of Humoral Immunity. Nat Commun (2015) 6:5997. 10.1038/ncomms6997 25609381

[13] Murakami M Kamimura D Hirano T. Pleiotropy and Specificity: Insights From the Interleukin 6 Family of Cytokines. Immunity (2019) 50:812–31. 10.1016/j.immuni.2019.03.027 30995501

[14] Kang S Tanaka T Narazaki M Kishimoto T. Targeting Interleukin-6 Signaling in Clinic. Immunity (2019) 50:1007–23. 10.1016/j.immuni.2019.03.026 30995492

[15] Zhang C Zhang M Qiu W Ma H Zhang X Zhu Z. Safety and Efficacy of Tocilizumab *Versus* Azathioprine in Highly Relapsing Neuromyelitis Optica Spectrum Disorder (TANGO): An Open-Label, Multicentre, Randomised, Phase 2 Trial. Lancet Neurol (2020) 19:391–401. 10.1016/S1474-4422(20)30070-3 32333897 PMC7935423

[16] Haas J Bekeredjian-Ding I Milkova M Balint B Schwarz A Korporal M. B Cells Undergo Unique Compartmentalized Redistribution in Multiple Sclerosis. J Autoimmun (2011) 37:289–99. 10.1016/j.jaut.2011.08.003 21924866

[17] Kemmerer C Pernpeintner V Ruschil C Abdelhak A Scholl M Ziemann U. Differential Effects of Disease Modifying Drugs on Peripheral Blood B Cell Subsets: A Cross Sectional Study in Multiple Sclerosis Patients Treated With Interferon-β, Glatiramer Acetate, Dimethyl Fumarate, Fingolimod or Natalizumab. PloS One (2020) 15:e0235449. 10.1371/journal.pone.0235449 32716916 PMC7384624

[18] Kowarik M Astling D Lepennetier G Ritchie A Hemmer B Owens G. Differential Effects of Fingolimod and Natalizumab on B Cell Repertoires in Multiple Sclerosis Patients. Neurotherapeutics (2021) 18:364–77. 10.1007/s13311-020-00975-7 PMC8116403 33258072

[19] Moura RA Quaresma C Vieira AR Goncalves MJ Polido-Pereira J Romao VC. B-Cell Phenotype and IgD-CD27- Memory B Cells are Affected by TNF-Inhibitors and Tocilizumab Treatment in Rheumatoid Arthritis. PloS One (2017) 12:e0182927. 10.1371/journal.pone.0182927 28886017 PMC5590747

[20] Jacobi A Reiter K Mackay M Aranow C Hiepe F Radbruch A. Activated Memory B Cell Subsets Correlate With Disease Activity in Systemic Lupus Erythematosus: Delineation by Expression of CD27, IgD, and CD95. Arthritis Rheum (2008) 58:1762–73. 10.1002/art.23498 18512812

[21] Claes N Fraussen J Vanheusden M Hellings N Stinissen P Van Wijmeersch B. Age-Associated B Cells With Proinflammatory Characteristics Are Expanded in a Proportion of Multiple Sclerosis Patients. J Immunol (Baltimore Md 1950) (2016) 197:4576–83. 10.4049/jimmunol.1502448 27837111

[22] Fraussen J Marquez S Takata K Beckers L Montes Diaz G Zografou C. Phenotypic and Ig Repertoire Analyses Indicate a Common Origin of IgDCD27 Double Negative B Cells in Healthy Individuals and Multiple Sclerosis Patients. J Immunol (Baltimore Md 1950) (2019) 203:1650–64. 10.4049/jimmunol.1801236 PMC6736705 31391234

[23] Kowarik M Astling D Gasperi C Wemlinger S Schumann H Dzieciatkowska M. CNS Aquaporin-4-Specific B Cells Connect With Multiple B-cell Compartments in Neuromyelitis Optica Spectrum Disorder. Ann Clin Trans Neurol (2017) 4:369–80. 10.1002/acn3.418 PMC5454399 28589164

[24] Nakayamada S Kubo S Yoshikawa M Miyazaki Y Yunoue N Iwata S. Differential Effects of Biological DMARDs on Peripheral Immune Cell Phenotypes in Patients With Rheumatoid Arthritis. Rheumatology (Oxf Engl) (2018) 57:164–74. 10.1093/rheumatology/kex012 28371836

[25] Jenks SA Cashman KS Zumaquero E Marigorta UM Patel AV Wang X. Distinct Effector B Cells Induced by Unregulated Toll-Like Receptor 7 Contribute to Pathogenic Responses in Systemic Lupus Erythematosus. Immunity (2018) 49:725–39.e726. 10.1016/j.immuni.2018.08.015 30314758 PMC6217820

[26] Sachinidis A Xanthopoulos K Garyfallos A. Age-Associated B Cells (ABCs) in the Prognosis, Diagnosis and Therapy of Systemic Lupus Erythematosus (SLE). Mediterr J Rheumatol (2020) 31:311–8. 10.31138/mjr.31.3.311 PMC7641025 33163863

[27] Mahmood Z Muhammad K Schmalzing M Roll P Dörner T Tony H. CD27-IgD- Memory B Cells are Modulated by *In Vivo* Interleukin-6 Receptor (IL-6R) Blockade in Rheumatoid Arthritis. Arthritis Res Ther (2015) 17:61. 10.1186/s13075-015-0580-y 25888920 PMC4415279

[28] Nicolas P Ruiz A Cobo-Calvo A Fiard G Giraudon P Vukusic S. The Balance in T Follicular Helper Cell Subsets Is Altered in Neuromyelitis Optica Spectrum Disorder Patients and Restored by Rituximab. Front Immunol (2019) 10:2686. 10.3389/fimmu.2019.02686 31803192 PMC6877601

[29] Fan X Jiang Y Han J Liu J Wei Y Jiang X. Circulating Memory T Follicular Helper Cells in Patients With Neuromyelitis Optica/Neuromyelitis Optica Spectrum Disorders. Mediators Inflamm (2016) 2016:3678152. 10.1155/2016/3678152 27057097 PMC4804098

[30] Chavele K Merry E Ehrenstein M. Cutting Edge: Circulating Plasmablasts Induce the Differentiation of Human T Follicular Helper Cells Via IL-6 Production. J Immunol (Baltimore Md 1950) (2015) 194:2482–5. 10.4049/jimmunol.1401190 PMC4356730 25681343

[31] Shen P Fillatreau S. Antibody-Independent Functions of B Cells: A Focus on Cytokines. Nat Rev Immunol (2015) 15:441–51. 10.1038/nri3857 26065586

[32] Roldan E Brieva JA. Terminal Differentiation of Human Bone Marrow Cells Capable of Spontaneous and High-Rate Immunoglobulin Secretion: Role of Bone Marrow Stromal Cells and Interleukin 6. Eur J Immunol (1991) 21:2671–7. 10.1002/eji.1830211105 1936115

[33] Cassese G Arce S Hauser AE Lehnert K Moewes B Mostarac M. Plasma Cell Survival is Mediated by Synergistic Effects of Cytokines and Adhesion-Dependent Signals. J Immunol (2003) 171:1684–90. 10.4049/jimmunol.171.4.1684 12902466

[34] Nurieva RI Chung Y Martinez GJ Yang XO Tanaka S Matskevitch TD. Bcl6 Mediates the Development of T Follicular Helper Cells. Science (2009) 325:1001–5. 10.1126/science.1176676 PMC2857334 19628815

[35] Rawlings DJ Metzler G Wray-Dutra M Jackson SW. Altered B Cell Signalling in Autoimmunity. Nat Rev Immunol (2017) 17:421–36. 10.1038/nri.2017.24 PMC5523822 28393923

[36] Makiyama A Chiba A Noto D Murayama G Yamaji K Tamura N. Expanded Circulating Peripheral Helper T Cells in Systemic Lupus Erythematosus: Association With Disease Activity and B Cell Differentiation. Rheumatology (Oxford) (2019) 58:1861–9. 10.1093/rheumatology/kez077 30879065

[37] Stefanski A Wiedemann A Reiter K Hiepe F Lino A Dörner T. Enhanced Programmed Death 1 and Diminished Programmed Death Ligand 1 Up-Regulation Capacity of Post-Activated Lupus B Cells. Arthritis Rheumatol (Hoboken NJ) (2019) 71:1539–44. 10.1002/art.40897 30919595

[38] Bodhankar S Galipeau D Vandenbark A Offner H. PD-1 Interaction With PD-L1 But Not PD-L2 on B-Cells Mediates Protective Effects of Estrogen Against EAE. J Clin Cell Immunol (2013) 4:143. 10.4172/2155-9899.1000143 24009988 PMC3760596

